# Simulation and Experimental Study on Parameter Optimization for the Glass Molding Process of Automotive Panoramic Roofs

**DOI:** 10.3390/ma19122662

**Published:** 2026-06-20

**Authors:** Ruili Wang, Hongyan Wang, Na Xiao, Zihao Hu, Wenjun Tong, Xiaohong Yang, Wuyi Ming

**Affiliations:** 1 Department of Engineering, Huanghe University of Science and Technology, Zhengzhou 450008, China; 201309111@hhstu.edu.cn (R.W.); 201608156@hhstu.edu.cn (N.X.); 2 Henan Key Laboratory of Intelligent Manufacturing of Mechanical Equipment, Zhengzhou University of Light Industry, Zhengzhou 450002, China; hongyanwang923@163.com; 3 Guangdong Provincial Key Laboratory of Digital Manufacturing Equipment, Guangdong HUST Industrial Technology Research Institute, Huazhong University of Science and Technology, Dongguan 523808, China; zihaohu41@gmail.com; 4 School of Aerospace Engineering, Huazhong University of Science and Technology, Wuhan 430074, China; tongwj@hust.edu.cn; 5 Hebi Institute of Engineering and Technology, Henan Polytechnic University, Hebi 458030, China

**Keywords:** panoramic roof, glass molding process, process analysis, forming quality, parameter optimization

## Abstract

The automotive panoramic roof exhibits a large-size and thin-wall geometry, with a length-to-thickness ratio approaching the thousand level. This geometric feature makes its forming quality highly sensitive to forming conditions. During the glass molding process, variations in temperature evolution, loading, and cooling parameters may lead to residual stress accumulation and springback deformation, thereby affecting dimensional accuracy and final forming quality. In this study, a full-process finite element model was established and combined with an L16(4^5) orthogonal design to investigate the effects of five key process parameters—heating temperature, holding time, quenching air velocity, quenching air pressure, and quenching time—on the mean residual stress and mean springback displacement in the glass molding process (GMP). The results showed that, within the given parameter ranges, heating temperature, holding time, and quenching time had relatively pronounced effects on the mean residual stress; the mean residual stress was relatively low when the heating temperature was 680 °C, the holding time was 3 s, and the quenching time was 12 s. Heating temperature, quenching air velocity, and quenching time had relatively pronounced effects on the mean springback displacement; the mean springback displacement was relatively low when the heating temperature was 677.5 °C, the quenching air velocity was 13 m/s, and the quenching time was 10 s. Based on the orthogonal analysis, regression models for the mean residual stress and mean springback displacement were further developed, and parameter combinations were screened using the NSGA-III method. Experimental validation showed that the relative error of the mean residual stress was controlled within 15%, indicating that the established model could, to some extent, capture the relationship between process parameters and forming quality indicators, thereby providing guidance for precision forming and process optimization of large-scale thin-walled automotive panoramic roofs.

## 1. Introduction

As a long-established inorganic material, glass has become a foundational component of modern material systems [1,2]. Owing to its excellent transparency, chemical stability, and processability, it has evolved into a key material supporting strategic emerging industries [3,4]. Glass is widely used in transportation (automobiles, rail transit, aviation) and consumer electronics, where ultra-thin, flexible variants promote foldable display technologies [5,6]. Additionally, it is extensively utilized in new energy and functional materials, including solar cell encapsulation, high-transmittance modules, and smart glass for dimming and thermal insulation [7,8]. As application scenarios expand, requirements for the structural design, functional integration, and manufacturing precision of glass continuously increase. In the automotive field, panoramic roofs are developing toward larger sizes and higher coverage ratios to enhance interior lighting, visual openness, and exterior design. However, this trend imposes significantly higher demands on the manufacturing processes and forming quality of automotive panoramic roofs.

Early studies on high-temperature glass forming were often based on the viscous-flow assumption, in which softened glass was generally approximated as a high-viscosity Newtonian fluid to describe its deformation response under gravity or mold constraints. Tuck et al. [9] and Stokes [10] applied the Newtonian fluid constitutive equation to simulate the bending forming process of viscous glass materials in a mold under gravity. Hunt [11] conducted a numerical analysis of the gravity sagging process of automotive glass, treated glass as a high-viscosity fluid material, derived the governing equation for viscosity variation with time, and obtained two-dimensional and three-dimensional deformation results for windshield glass. As research continued to advance, international scholars gradually recognized that glass in the high-temperature forming stage was not simply a viscous fluid, but exhibited distinct temperature-dependent viscoelastic characteristics. Parsa et al. [12] defined automotive glass as a viscoelastic material and used two finite element software packages to simulate, compare, and evaluate the creep forming of automotive windshield glass. Ananthasayanam et al. [13] further established a temperature-dependent viscoelastic model based on a Prony series, and their simulation results agreed well with experiments, with dimensional deviation controlled within 0.5 μm. Hung et al. [14] focused on stress relaxation during the hot bending forming stage and structural relaxation during the cooling stage, and analyzed the effects of process parameters on the stress and dimensional accuracy of the finished glass. Shutov et al. [15,16,17] showed that key heat-treatment parameters, including hardening temperature, heat transfer coefficient, glass thickness, and cooling duration, significantly affect final glass performance. Vu et al. [18] investigated, through simulation, the effects of parameters such as the friction coefficient and blank-holder force on thickness distribution, local thinning, and edge wrinkling during the deep drawing forming of glass. Overall, international research has evolved from early fluid-based approximations to more systematic studies addressing high-temperature viscoelastic constitutive behavior, heat-treatment processes, interfacial effects, and forming-quality control.

With the continued advancement of related research, numerical simulations of glass forming have gradually extended to thermo-mechanical coupling analyses, with attention to heat transfer, residual-stress evolution, springback behavior, and process optimization. Corre et al. [19] simulated the two-dimensional glass slumping process based on the finite element method, analyzed the effects of radiative heat transfer on temperature distribution and forming accuracy, and pointed out that neglecting radiative heat transfer would lead to large errors. Nielsen et al. [20] established and validated a three-dimensional numerical model of the glass tempering process, which could accurately predict the distributions of transient stress and residual stress in complex three-dimensional glass components. Zhang [21] investigated the forming of automotive rear windshield glass, revealed the evolution of forming springback and tempering stress, and improved forming accuracy through displacement compensation and process parameter optimization. Iglesias et al. [22] proposed a numerical method for predicting nonuniform residual stress during glass tempering and indicated that accounting for nonuniform heat transfer caused by local flow was important for improving the prediction accuracy of residual stress. Fu et al. [23] improved the prediction of residual stress by considering nonuniform heat transfer and residual-stress relaxation under different molding conditions. Chen et al. [24] studied the molding quality of automotive irregular-shaped glass and pointed out that forming temperature and forming pressure were the key factors affecting residual stress and dimensional accuracy, respectively. Yang et al. [25] further investigated the effects of forming temperature and pressure on shape deviation, cracks, and surface quality during the molding of ultra-thin glass, and pointed out that forming temperature was an important factor affecting forming defects and surface quality. Zhao et al. [26] combined numerical simulation with NSGA-II to perform multi-objective optimization of process parameters for 3D curved glass, providing an effective strategy for simultaneously controlling stress and shape deviation.

In terms of material-parameter acquisition, Yao et al. [27] experimentally established the high-temperature viscoelastic parameters of glass and pointed out that the high-temperature elastic modulus had an important effect on forming simulation accuracy. Shu et al. [28] established a viscoelastic constitutive model of high-refractive-index lanthanum optical glass based on compression creep tests and introduced a structural relaxation model into the annealing simulation of precision glass lens molding. Their results showed that considering structural relaxation yielded more accurate prediction of aspheric lens profile deviation than the conventional thermal expansion model. Luo et al. [29] analyzed the effect of interfacial friction on glass filling behavior through simulation and experiment. The results showed that friction in the groove contact region had an important effect on the filling performance. Chen et al. [30] studied heat-transfer strategies and energy efficiency in the molding process of large-size automotive instrument glass, established a heat-transfer model for a metal heating plate–thermal-conductive plate mold system, and pointed out that optimizing heat flux density could reduce heating energy consumption while ensuring forming quality. Huang et al. [31] established a viscoelastic glass molding model based on thermo-structural coupling analysis and combined neural networks with a genetic algorithm to conduct multi-objective optimization of process parameters. Related research has gradually expanded from analysis of a single forming process to multiple aspects, including material-parameter characterization, interfacial contact description, boundary-condition setting, and full-process coupled modeling, thereby laying a foundation for improving the simulation accuracy and engineering applicability of the glass molding process (GMP).

Beyond numerical simulation of glass forming, optimisation of automotive component manufacturing has become essential for improving product quality and production efficiency. Advanced strategies such as surrogate modelling, machine learning, and multi-objective evolutionary algorithms have been widely applied to reduce defects and enhance structural performance in automotive manufacturing [32]. For example, Chai et al. [33] proposed a multistage heuristic optimisation framework combining inverse simulation and Hooke–Jeeves local search, which reduces the computational cost of simulation-based optimisation. Purr et al. [34] developed an inline optimisation method for car body stamping using support vector machines and linear regression to recommend process parameters based on historical production data. Wang et al. [35] integrated a neural network prediction model with a genetic algorithm to achieve intelligent parameter recommendation in automotive steering wheel manufacturing. At the design stage, Jankovics et al. [36] combined topology optimisation with additive manufacturing constraints to realise lightweight design of automotive structural components. Inspired by these simulation-based and data-driven optimisation approaches, the application of systematic multi-objective evolutionary algorithms to the GMP of large-scale panoramic roofs is necessary for balancing residual stress and springback displacement.

Based on the above research status, existing studies have mainly focused on ordinary automotive glass, optical glass, or analyses of local forming stages, whereas research on the evolution of residual stress and springback displacement throughout the full GMP of large-size automotive panoramic roof glass remains relatively limited. In particular, under continuous operating conditions involving heating, forming, quenching, and slow cooling, the coupled effects of different process parameters on the final forming quality of glass still lack systematic analysis. Therefore, this study took automotive panoramic roof glass as the research object, established a full-process finite element model for the GMP, investigated the effects of key process parameters on the mean residual stress and mean springback displacement using an orthogonal experimental design, and further analyzed and verified the relatively optimal parameter combinations, with the aim of providing a reference for improving the GMP of automotive panoramic roofs.

## 2. Experimental and Material Testing Equipment

### 2.1. Glass Molding Experimental Equipment

**Figure 1:**
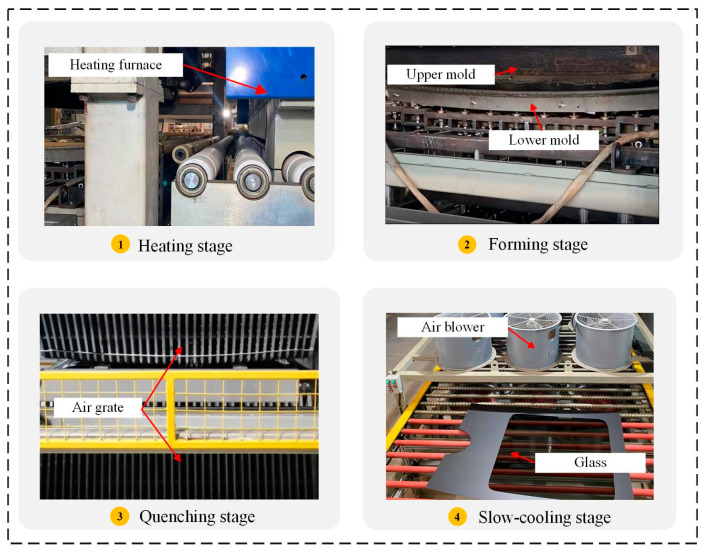
The flow of the GMP for panoramic roofs.

The molding process of glass is mainly achieved through hot-press technology [37]. According to differences in glass size specifications and geometric shapes, GMP is mainly divided into two types: sagging forming and press forming [38]. In this study, the equipment and process flow were analyzed based on actual production conditions. The entire forming system mainly consisted of a heating device, a press-forming device, an air-grid quenching device, and a subsequent slow-cooling device. These devices jointly completed the full process of glass heating and softening, press forming, cooling, and shape fixing, as shown in Figure 1. The material used in the present study was soda-lime silicate glass, which is the most widely used material in automotive glass applications. Its main chemical composition consists of silicon dioxide, calcium oxide, sodium oxide, magnesium oxide, and aluminum oxide. The glass samples were provided by an industrial partner, an automotive glass manufacturer, and were representative of the material used in the actual production line for automotive panoramic roofs. Therefore, this material selection ensures that the simulation and process analysis are consistent with industrial manufacturing conditions. During the heating stage, the glass was heated at a preset furnace temperature of 680 °C for 200 s. Then, the glass entered the press-forming stage under high-temperature conditions. The upper mold applied downward loading for 2 s, followed by a pressure-holding process of 0.8 s after forming to stabilize the curved shape. Subsequently, the mold moved upward within 2 s to complete withdrawal. After forming, the glass immediately entered the tempering and quenching stage. This stage mainly relied on the air-grid quenching device to apply forced cooling to the glass surface. Under the process conditions selected in this study, a wind pressure of 3 MPa was applied for 10 s of quenching, rapidly reducing the glass surface temperature to approximately 150 °C. During this process, the air-grid device not only affected the cooling rate but was also directly related to the formation of temperature gradients between the glass surface and interior, thereby exerting an important influence on residual stress distribution and subsequent springback deformation. After quenching, the glass entered the slow-cooling stage, in which it was continuously cooled for 200 s using the slow-cooling device until the temperature gradually decreased to approximately room temperature (25 °C), thereby promoting the release of residual thermal stress and ensuring dimensional stability of the finished product.

### 2.2. Modulus Testing Equipment and Test Results

**Figure 2:**
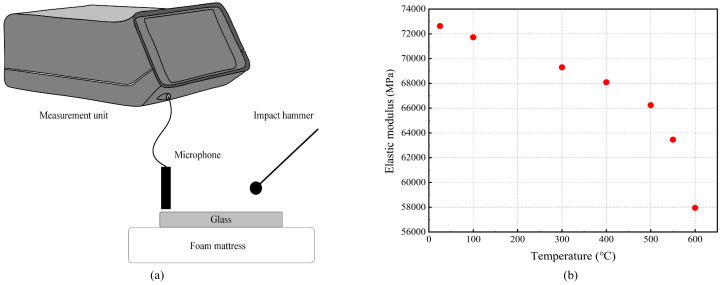
Experimental setup and test results for elastic modulus measurement of glass: (**a**) Modulus testing equipment; (**b**) Elastic modulus test results of glass.

In this study, the elastic modulus of the glass material was measured using the GrindoSonic MK7 elastic modulus tester (GrindoSonic BV, Leuven, Belgium). As shown in Figure 2a, this instrument uses pulse excitation technology to determine the dynamic elastic modulus based on the vibrational response of the specimen. Because this method did not require destructive loading, it could be considered a nondestructive testing method for modulus characterization. The device operates over a frequency range of 20 Hz to 150 kHz, with a reference accuracy better than 0.005%, providing a reliable basis for elastic modulus measurement. Using this tester, glass specimens were tested at discrete temperature points of 25, 100, 300, 400, 500, 550, and 600 °C, and the corresponding elastic modulus values were obtained, as shown in Figure 2b. By comparing and analyzing the results at different temperature points, the temperature-dependent variation in the dynamic elastic modulus of the glass material can be evaluated, providing experimental support for subsequent modulus fitting and model optimization.

## 3. Simulation Modeling of the GMP

In this study, a thermo-mechanical coupled finite element model of the full GMP of automotive panoramic roofs was established based on Abaqus/CAE 2022 (Dassault Systèmes, Vélizy-Villacoublay, France) and solved using the Abaqus/Standard 2022 solver. The model consisted of the glass, upper mold, and lower mold, and sequentially considered the process stages of heating, mold closing and forming, pressure holding, mold opening, quenching, and slow cooling. To accurately capture the thermo-mechanical behavior of the glass during the molding process, coupled temperature–displacement analysis was adopted for the heating, quenching, and slow-cooling stages, while viscous analysis was employed during the forming stage. Geometric nonlinearity was considered throughout the simulation. By defining the geometric structure, material properties, boundary conditions, and process parameters, numerical analysis was conducted on stress evolution, springback deformation, and residual stress formation during the glass forming process.

### 3.1. Geometric Model and Dimensions

**Figure 3:**
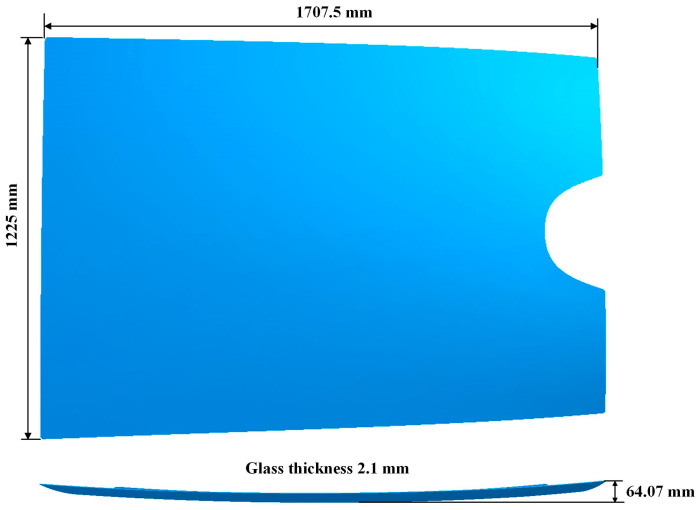
Geometric dimensions of the glass.

In mass production, automotive panoramic roof glass commonly adopts a laminated structure to satisfy safety and comfort requirements. However, the viscoelasticity of the interlayer and its coupling with the glass interface increase the complexity of thermo-mechanical analysis and numerical modeling in GMP. Therefore, to simplify the model while retaining analytical validity, this study focuses on the forming behavior of a single-piece panoramic roof glass during GMP. Figure 3 illustrates the geometric model used in the simulation. The glass has a width of 1225 mm, a length of approximately 1707.5 mm, a depth of 64.07 mm, and a thickness of 2.1 mm.

**Figure 4:**
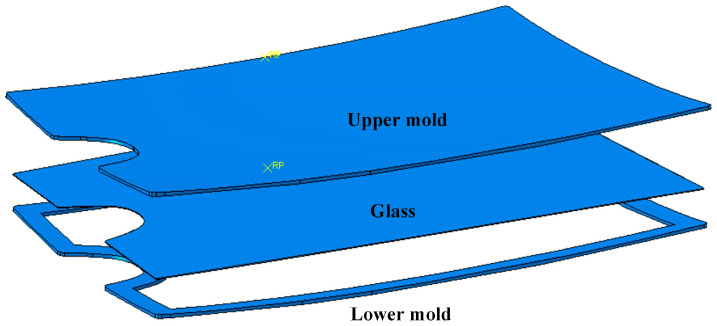
Finite element assembly for the GMP of a panoramic roof.

Since this study mainly focused on the contact interaction between the glass and the working surfaces of the molds during forming, as well as the corresponding deformation response, the primary role of the molds in the model was to provide surface constraints and transfer contact loads. Therefore, while ensuring accurate representation of the geometric features of the mold working surfaces and the contact boundaries, the mold thickness and some noncritical structures were appropriately simplified to reduce model complexity and improve solution efficiency. Only a finite element assembly consisting of the upper mold, flat glass sheet, and lower mold was established, as shown in Figure 4.

### 3.2. Material Properties

In numerical simulation, the definition of material properties is one of the fundamental steps in model development and directly determines the reliability of the predicted temperature-field transfer, structural mechanical response, residual stress, and springback. In terms of the material model, the thermophysical and mechanical parameters of glass listed in Table 1 were applied as input and served as the unified material basis for the subsequent coupled analysis of the temperature field and stress field, thereby ensuring the comparability and repeatability of the simulation results under different process parameter conditions. Considering that the stiffness of the mold material was much higher than that of glass and that the effect of mold deformation on the forming results could be approximately neglected within the scope of this study, the molds were modeled as discrete rigid bodies to reduce the number of computational degrees of freedom and improve solution efficiency. Under this modeling strategy, the molds did not participate in elastic deformation calculation; therefore, no additional deformation-related material-property parameters needed to be defined.

**Figure 5:**
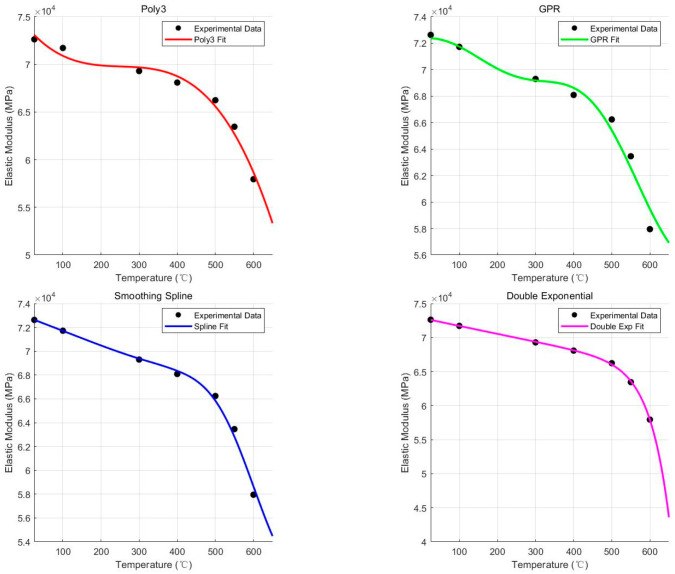
Fitting results of the testing instrument data.

For the data obtained from the testing instrument, four fitting methods were utilized in this study: cubic polynomial fitting, Gaussian process regression, smoothing spline fitting, and double exponential function fitting. The results are shown in Figure 5. As shown in Table 2, the cubic polynomial model had an R^2^ of 0.9807, an RMSE of 655.33, and an MAE of 638.39, indicating that it could reasonably reflect the overall trend of the data. The Gaussian process regression model yielded R^2^ = 0.9731, RMSE = 772.66, and MAE = 595.17. Its fitted curve was relatively smooth, and the mean error was slightly lower; however, noticeable deviations still occurred near some measurement points. The smoothing spline model achieved R^2^ = 0.9928, RMSE = 400.95, and MAE = 297.98, indicating that it provided a good balance between capturing the overall trend and controlling local errors. The double exponential function model showed the best performance, with an R^2^ of 0.9996 and RMSE and MAE values of only 96.70 and 71.87, respectively, and could accurately reproduce the nonlinear variation reflected in the experimental data.

**Figure 6:**
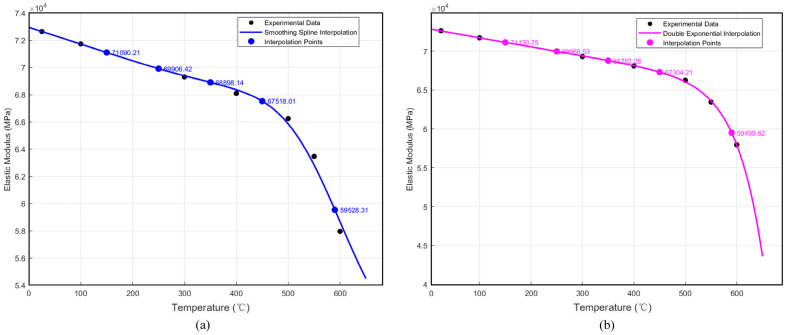
Comparison of elastic modulus interpolation results: (**a**) Smoothing spline; (**b**) Double exponential.

After the above experimental testing and fitting analysis were completed, a continuous functional relationship between the elastic modulus of glass and temperature was obtained, providing a basis for characterizing material stiffness over a wider temperature range. Based on the comparison of the fitting results, the double exponential function model showed relatively favorable statistical performance, and its variation trend was also generally consistent with the temperature-dependent mechanical behavior of glass [39,40]. The measured modulus data indicated that the elastic modulus of glass decreased nonlinearly with increasing temperature, and this decrease became more evident in the higher-temperature range. Compared with the other fitting forms, the double exponential function provided relatively good curve continuity within the measured temperature range and could describe the accelerated decrease in elastic modulus at elevated temperatures in a reasonably smooth manner. Therefore, within the experimentally measured temperature range, the double exponential fitting method could be considered a relatively suitable empirical approach for interpolating the temperature-dependent elastic modulus and defining material parameters for finite element simulation. Although the existing experimental data basically covered the main temperature range considered in this study, engineering simulation and process evaluation may still require additional material-parameter data within the measured range to support the prediction of high-temperature forming behavior and material response under extreme service conditions. In this study, the modulus values predicted by the elastic modulus tester were used as interpolation inputs, as shown in Figure 6, and their applicability and degree of influence were further verified in the subsequent numerical simulation. The simulation accuracy of the three methods was compared based on the average relative error at each measurement point. The simulation accuracy was evaluated by comparing the simulated residual stresses with the experimentally measured edge-stress values at selected representative measurement points. The relative errors at these points were first calculated and then averaged, and the simulation accuracy was defined as 100% minus this average relative error. Without fitting, the simulation accuracy was 72.64%, indicating that a certain deviation still existed between the original simulation data and the experimental results. After smoothing spline fitting was adopted, the simulation accuracy increased to 81.62%, indicating that this method could improve the consistency between the simulation and experimental results to some extent. In contrast, after double exponential fitting, the simulation accuracy further increased to 86.38%, showing a more significant improvement and indicating that this method more reasonably characterized the variation law of material parameters. Based on the above results, the elastic modulus supplementary values predicted by double exponential fitting were selected as material-parameter inputs in the subsequent simulations to improve the rationality of parameter definition and enhance the reliability of the subsequent analysis results. Table 3 lists the elastic modulus values of glass at different temperatures in the range of 25 °C to 600 °C.

Glass exhibits pronounced time-dependent behavior during GMP. Therefore, the generalized Maxwell model was selected in this study to describe the viscoelastic behavior of automotive panoramic roof glass during forming. This model can effectively capture the stress relaxation behavior and deformation evolution of glass during loading and holding stages; therefore, it is suitable for constitutive analysis and finite element simulation of the GMP. In addition, the generalized Maxwell model can be implemented in Abaqus through Prony series parameter input, providing reliable constitutive support for subsequent simulation analysis. In combination with the research content of this study and the available research conditions, the model parameters were determined with reference to glass creep test data reported in the literature, as listed in Table 4.

### 3.3. Boundary Conditions

In this study, the heating stage adopted a coupled temperature–displacement transient analysis. Heat conduction within the glass was considered in the transient thermal analysis, where the temperature field evolution inside the glass was described within the finite element formulation of the heat transfer problem. The heat-transfer process was described using a film condition on the glass surface, with a film coefficient of 0.12 mW/(mm^2^·K). The heating environment temperature was set to 680 °C, the initial glass temperature was set to 25 °C, and the analysis time was 200 s. No additional mechanical loads or constraints were applied in this stage. The GMP stage consisted of three analysis steps: mold closing and forming, pressure holding, and mold opening. The upper mold moved downward and closed with the lower mold within 2 s, then remained in position for 0.8 s to achieve pressure holding, and finally returned upward within 2 s to complete mold opening. The entire stage lasted 4.8 s, and the downward displacement of the upper mold was 68.6 mm. To simulate thermo-mechanical interactions during forming, a surface-to-surface contact with finite sliding was defined between the glass and molds, using a penalty friction model with a coefficient of 0.01; all other contact settings were kept as default. The molds were modeled as discrete rigid bodies controlled via reference points. The lower mold RP was fully constrained, while the upper mold RP was constrained in the X and Y directions, with the Z-direction released to impose the prescribed forming displacement. Subsequently, quenching and slow-cooling processes were carried out, both of which used surface film conditions to describe heat transfer. In these stages, heat exchange between the glass and the surrounding environment is modeled using a combined surface boundary condition accounting for convection and radiation, where convective heat transfer is described by a heat transfer coefficient based on Newton’s law of cooling, and radiative heat transfer is represented using an equivalent linearized radiative heat-transfer coefficient. The cold-source temperature was set to the air-grid temperature of 25 °C, and the film coefficient in the quenching stage was 385.195 W/(m^2^·K). In the slow-cooling stage, the convective heat-transfer coefficient was calculated using the same procedure as that used in the quenching stage and was 60.76 W/(m^2^·K). The surface radiative heat-transfer coefficient was 9.31 W/(m^2^·K), giving an overall heat-transfer coefficient of 70.07 W/(m^2^·K).

**Figure 7:**
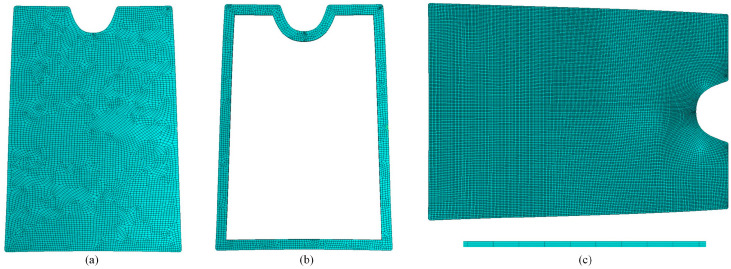
Mesh generation: (**a**) mesh of the upper mold; (**b**) mesh of the lower mold; (**c**) mesh of the glass.

Mesh generation is an important step in finite element simulation, and the mesh quality and element type directly affect the accuracy and convergence of the temperature-field and stress-field calculations. Because the molds mainly served as forming constraints and heat-transfer boundaries, and their stiffness was substantially higher than that of the glass, mold deformation during forming and thermal loading was assumed to have a limited influence on the simulation results. Accordingly, the molds were modeled using R3D4 rigid elements to reduce the computational degrees of freedom and improve solution efficiency and numerical stability. The meshing results of the upper and lower molds are shown in Figure 7a and Figure 7b, respectively. The mesh size of the upper mold was 15 mm, with a total of 19,591 elements, while the mesh size of the lower mold was 10 mm, with a total of 10,215 elements. This mesh configuration balanced computational accuracy and solution efficiency and met the requirements of the subsequent simulation analysis. The panoramic roof glass was meshed using C3D8T elements. This element is an eight-node thermally coupled hexahedral element that can simultaneously represent the displacement and temperature fields, meeting the requirements for coupled temperature-field and stress-field analysis throughout the full GMP. Three layers were assigned in the thickness direction, which helped capture the temperature gradient through the thickness and the resulting stress-gradient variation while achieving a reasonable balance between computational accuracy and efficiency. To further verify the rationality of the selected glass mesh size, a mesh convergence study was performed using 12 mm, 10 mm, 8 mm, and 6 mm global mesh sizes. The average errors of edge residual stress were 26.55%, 18.96%, 13.62%, and 10.96%, respectively. The results showed that the error decreased significantly when the mesh size was refined from 12 mm to 8 mm, whereas further refinement from 8 mm to 6 mm only reduced the error from 13.62% to 10.96%, indicating limited accuracy improvement but a considerable increase in computational cost. Therefore, the 8 mm mesh was selected for the glass model. Under the selected mesh size of 8 mm, the glass was divided into a total of 82,392 elements, as shown in Figure 7c.

### 3.4. Experimental Scheme Design

Table 5 lists the control factors and their corresponding level settings in the experiment, including heating temperature (*A*), holding time (*B*), quenching air velocity (*C*), quenching air pressure (*D*), and quenching time (*E*). These factors are important process parameters affecting the quality of the panoramic roof, and their variations have different degrees of influence on the mean residual stress and mean springback displacement. To ensure the comprehensiveness of the experimental design and the representativeness of the data, four levels were set for each factor, covering the typical variation ranges of each factor in actual production. Table 6 presents the specific arrangement of the 16 orthogonal experimental groups, with each group corresponding to a different parameter combination. If a full-factorial experimental method were adopted to investigate five factors and four levels, the number of experiments would reach 1024, which would require substantial experimental cost and could introduce considerable data dispersion during the experimental process, making it difficult to identify the optimal parameter combination. In contrast, using the L16(4^5) orthogonal optimization method required only 16 experiments to effectively screen relatively optimal parameter combinations, significantly reducing the number of experiments and resource consumption. Through this orthogonal experimental design method, the effects of different factors on the mean residual stress and mean springback displacement during glass forming could be effectively analyzed and compared, providing a scientific basis for subsequent forming-process optimization.

## 4. Simulation Results and Analysis

### 4.1. Quenching Stage Results

**Figure 8:**
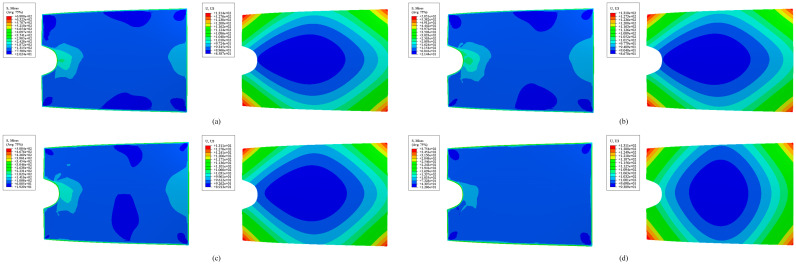
Comparison of quenching simulation results for the No. 1 to No. 4 simulations: (**a**) the first simulation; (**b**) the second simulation; (**c**) the third simulation; (**d**) the fourth simulation.

The quenching simulation results for the simulations from No. 1 to No. 4 are shown in Figure 8. Under the condition that the heating temperature was kept constant at 672.5 °C, the stress field and U3 displacement field of the glass during quenching exhibited certain variation characteristics as the process parameter combinations were gradually adjusted. The stress results showed that high-stress regions were mainly distributed in geometrically discontinuous areas, such as the groove transition zones and edges, which may have been associated with geometric discontinuity and non-uniform thermal gradients during the quenching process. These regions experienced higher cooling rates, which could have led to local thermal shrinkage mismatch and stress concentration. From the No. 1 to No. 4 simulation, the overall stress level showed a decreasing trend. In particular, the stress range of the No. 4 simulation was 12.86–375.4 MPa, indicating that under the current parameter combination, local stress concentration may have been alleviated to some extent, and the uniformity of the internal response of the glass was improved compared with those in the previous simulations. Meanwhile, the U3 displacement results showed that the maximum displacement in each simulation remained basically constant at approximately 131 mm, indicating that the overall variation in the high-displacement regions was relatively limited under different working conditions. In contrast, the minimum displacement gradually increased from 85.87 mm in the No. 1 simulation to 93.89 mm in the fourth simulation. This phenomenon indicated that, during the quenching stage, the response in low-displacement regions, such as the central area of the glass, became more pronounced. This variation may be related to changes in the temperature gradient within the quenching stage, which could influence the deformation coordination between different regions of the glass and lead to variations in local shrinkage behavior. In addition, the release of structural constraints after mold opening may have resulted in local deformation adjustment, producing a mild tendency of displacement springback in the central region.

**Figure 9:**
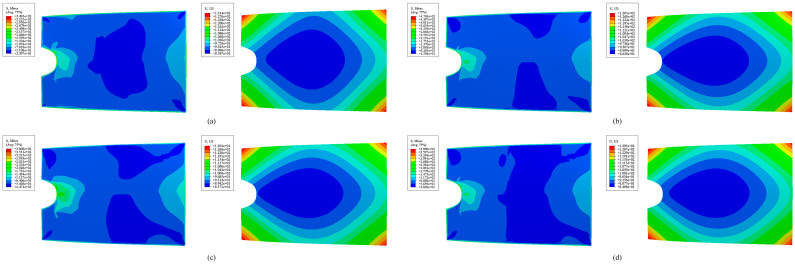
Comparison of quenching simulation results for the No. 5 to No. 8 simulations: (**a**) the fifth simulation; (**b**) the sixth simulation; (**c**) the seventh simulation; (**d**) the eighth simulation.

The quenching simulation results for the simulations from No. 5 to No. 8 are shown in Figure 9. Under these parameter combinations, the stress field and U3 displacement field of the glass during quenching also exhibited certain variation characteristics. The stress results showed that high-stress regions were still mainly distributed in geometrically discontinuous areas, such as the groove transition zones and edges, indicating that the local geometric transition regions of the component remained sensitive areas for stress response under different working conditions. Taking the No. 5 simulation as an example, its stress range was 23.97–349.2 MPa, indicating that the overall stress level under this working condition was relatively low and that local stress concentration may have been relatively weak. This behavior may be associated with the fact that the component had already undergone significant geometric stabilization during the forming stage, which could reduce the sensitivity of the stress response to subsequent parameter variations during quenching. Meanwhile, the U3 displacement results showed that the maximum displacement in each simulation remained basically within the range of 130.3–131.4 mm, whereas the minimum displacement fluctuated slightly between 84.98 and 86.3 mm. This phenomenon indicated that the parameter adjustments from the No. 5 to No. 8 simulations may have had a relatively limited effect on the overall displacement distribution of the glass, and that the changes during the quenching stage were mainly reflected in slight local adjustments. These small variations may be attributed to slight differences in cooling conditions and boundary constraints during quenching, which could lead to minor adjustments in local deformation behavior.

**Figure 10:**
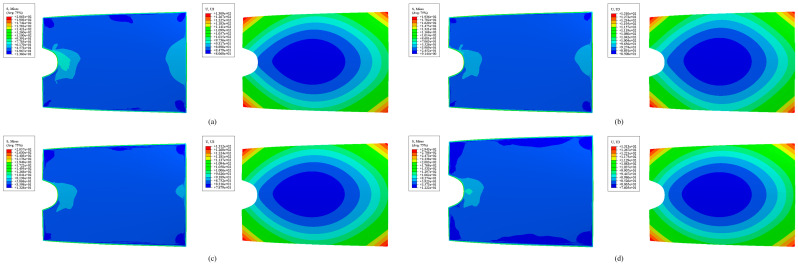
Comparison of quenching simulation results for the No. 9 to No. 12 simulations: (**a**) the ninth simulation; (**b**) the tenth simulation; (**c**) the eleventh simulation; (**d**) the twelfth simulation.

Figure 10 shows the quenching simulation results for the simulations from No. 9 to No. 12. Under different parameter combinations, certain differences were observed in both the stress field and the U3 displacement field of the glass during the quenching stage. Among them, the residual stress range of the tenth simulation was 9.144–193.6 MPa, indicating that the overall stress level under this condition was relatively low and that local stress concentration was relatively weak. This reduction in stress level may be associated with more balanced heat transfer and improved compatibility of boundary heat exchange conditions, which led to a smoother temperature gradient distribution and suppressed excessive thermal stress accumulation. Meanwhile, the U3 displacement results showed that the maximum displacement from the No. 9 to No. 12 simulations remained basically stable within the range of 130.9–131.3 mm, whereas the minimum displacement fluctuated between 76.05 and 85.08 mm. This indicated that different parameter combinations had little effect on the overall contour of the glass, but did affect the degree of deformation in local regions along the U3 direction. This was mainly because the cooling of the upper and lower surfaces of the glass, as well as through the thickness direction, was not synchronized during quenching, resulting in differences in shrinkage among different regions. The underlying mechanism is that differences in cooling rates along the thickness direction and between the upper and lower surfaces persist during quenching, resulting in different degrees of coordination in stress relaxation and shrinkage between the interior and outer regions. Meanwhile, boundary constraints further intensify the non-uniform deformation in local regions. When a local region cooled more rapidly or was subjected to stronger constraint, its shrinkage or warping response along the U3 direction became more pronounced, which was reflected in the fluctuation of the minimum displacement. In contrast, the relatively small change in the maximum displacement indicated that the overall shape of the glass remained basically stable during the quenching stage.

**Figure 11:**
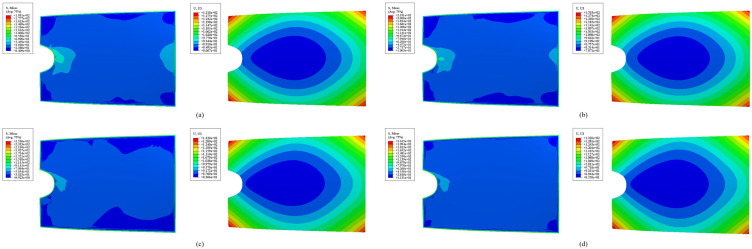
Comparison of quenching simulation results for the No. 13 to No. 16 simulations: (**a**) the thirteenth simulation; (**b**) the fourteenth simulation; (**c**) the fifteenth simulation; (**d**) the sixteenth simulation.

The quenching simulation results for the No. 13 to No. 16 simulations are shown in Figure 11. Under different parameter combinations, the stress field and U3 displacement field of the glass during the quenching stage also exhibited certain differences. Among them, the residual stress range of the No. 13 simulation was 8.409–193.1 MPa, indicating that the overall stress level under this condition was relatively low and that local stress concentration was relatively weak. This may be associated with a relatively uniform temperature distribution during the cooling process, which could reduce the temperature gradient between the surface and the interior, thereby weakening the non-uniform thermal shrinkage and, to some extent, alleviating stress accumulation. Meanwhile, the U3 displacement results showed that the maximum displacement from the thirteenth to the No. 16 simulations remained basically stable within the range of 131.8–132 mm, whereas the minimum displacement varied between 78.71 and 85.58 mm. This indicates that different parameter combinations still have little effect on the overall contour of the glass, but do have a certain influence on the degree of deformation in local regions along the U3 direction. This behavior suggests that global deformation is mainly governed by boundary constraints, whereas local variations are likely related to non-uniform cooling during quenching and the resulting spatial differences in thermal shrinkage. Different parameter combinations may further alter local heat transfer and constraint conditions, thereby contributing to variations in residual stress and local displacement response.

### 4.2. Effects of Process Parameters on Residual Stress

**Figure 12:**
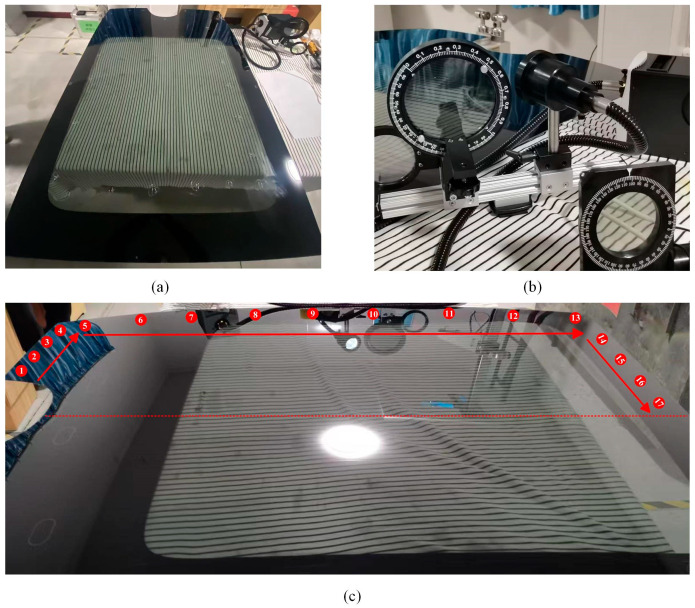
Panoramic roof and residual stress measurement setup: (**a**) panoramic roof specimen; (**b**) VRP-100 edge stress meter for black-edge glass; (**c**) arrangement of residual stress measurement points.

This study focused on a panoramic roof glass for new-energy electric vehicles provided by a company research and development center, as shown in Figure 12a. To obtain the residual stress and springback behavior after glass forming, a VRP-100 edge stress meter (Strainoptics, Burnsville, MN, USA) for black-edge glass was used for stress measurement, as shown in Figure 12b. The instrument adopts a reflective non-contact measurement method and is suitable for stress detection in black-edge glass edge regions. Based on the residual stress analysis requirements, 17 measurement points were arranged along the glass edge region, as shown in Figure 12c. Among them, nine points were distributed along the upper edge in the transverse direction, while four points were arranged along each side edge in the longitudinal direction to characterize stress variations in different boundary regions. Based on these locations, eight representative points (Points 1, 3, 5, 6, 8, 10, 12, and 15) were further selected for residual stress extraction, and the mean residual stress was calculated for subsequent result evaluation and comparative analysis under different process parameter conditions. For the springback behavior, seven representative points were selected along the glass length direction from the edge region to the central region in areas with relatively continuous displacement variation. The springback displacement at each point was defined as the difference in the U3 displacement component between the end of the molding stage and the end of the slow-cooling stage. The springback displacements of all selected points were then statistically processed to obtain the mean springback displacement, which was used as the statistical index for springback evaluation and comparative analysis under different process conditions.

Table 7 summarizes the mean residual stress and mean springback displacement results of the formed glass under the 16 orthogonal simulation schemes. In terms of response distribution, the mean residual stress varied within the range of 20.48–33.18 MPa, while the mean springback displacement fluctuated within the range of 2.715–11.149 mm. These results indicated that the formation processes of residual stress and springback response may have corresponded to different dominant factors and action paths. Their variations were not only affected by individual parameters, but may also have been related to temperature-field evolution, viscoelastic relaxation of the material, and multi-parameter coupling effects. These results provided a data basis for the subsequent quantitative analysis of the effects of forming parameters on the mean residual stress and mean springback displacement.

The effects of different forming parameters on the residual stress of automotive panoramic roof glass were investigated through orthogonal experimental design and Minitab analysis. Table 8 presents the mean response of residual stress for each control factor. According to the range analysis results, the range value of heating temperature (*A*) was 8.28, which was relatively large among all factors, indicating that, within the scope of this study, it had a more pronounced effect on the mean residual stress. The range value of holding time (*B*) was 3.23, suggesting that it also had a certain influence on the mean residual stress, which may have been related to changes in the degree of internal stress relaxation in the glass during the holding stage. Quenching time (*E*) also showed a certain effect on residual stress, whereas the range values of quenching air velocity (*C*) and quenching air pressure (*D*) were relatively small, indicating that their effects were relatively limited. Overall, heating temperature (*A*), holding time (*B*), and quenching time (*E*) had more pronounced effects on the mean residual stress, while the effects of quenching air velocity (*C*) and quenching air pressure (*D*) were relatively weak.

**Figure 13:**
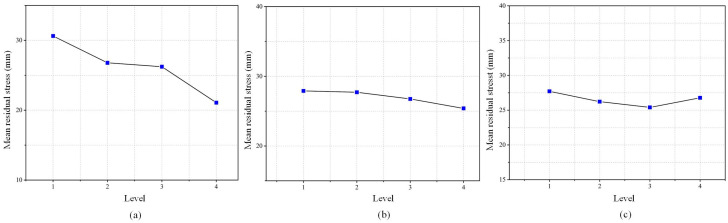
Main effects plot of process parameters on the mean residual stress of glass: (**a**) heating temperature; (**b**) holding time; (**c**) quenching time.

As shown in Figure 13a, the mean residual stress generally decreased with increasing heating temperature. At 672.5 °C, the mean residual stress was 30.62 MPa (No. 3). When the temperature increased to 675 °C, the mean residual stress decreased to 26.78 MPa (No. 7), indicating that the softening degree of the glass increased after heating and that the residual stress was released to a certain extent. As the temperature further increased to 677.5 °C, the mean residual stress further decreased to 26.23 MPa (No. 12). Although the decrease was smaller than that at 675 °C, it still indicated that increasing temperature promoted the reduction of residual stress to some extent. Finally, when the temperature increased to 680 °C, the mean residual stress decreased to 21.08 MPa (No. 16), indicating that at a higher temperature, the softening effect of the glass was further enhanced and the residual stress in the glass was more fully released.

As shown in Figure 13b, the mean residual stress generally decreased with increasing holding time, with only a slight change in the early stage followed by a gradual decrease. When the holding time was 0.5 s, the mean residual stress was 27.91 MPa (No. 9). When the holding time increased to 0.8 s, the mean residual stress slightly decreased to 27.71 MPa (No. 6), showing only a small change. This may have been because the increase in holding time from 0.5 s to 0.8 s was relatively small, and its regulation effect on the internal temperature field and stress state of the glass was not yet obvious; therefore, the change in residual stress was limited. As the holding time further increased to 1.5 s, the mean residual stress decreased to 26.75 MPa (No. 11), indicating that appropriately extending the holding time may have helped make the internal temperature of the glass more uniform and promoted the release of residual stress to some extent. Finally, when the holding time reached 3 s, the mean residual stress decreased to 25.4 MPa (No. 8), indicating that the residual stress still showed a decreasing trend under a longer holding time. This may have been related to the gradual enhancement of relaxation behavior of the glass at high temperature during the holding process, which further reduced the residual stress.

As shown in Figure 13c, the mean residual stress generally showed a trend of first decreasing and then slightly increasing with increasing quenching time. When the quenching time was 8 s, the mean residual stress was 27.71 MPa (No. 6). As the quenching time increased to 10 s, the mean residual stress decreased to 26.23 MPa (No. 12), indicating that the residual stress was reduced after appropriately extending the quenching time. When the quenching time was further increased to 12 s, the mean residual stress continued to decrease to 25.4 MPa (No. 8), indicating that, within this stage, extending the quenching time may have been beneficial for further adjustment of the stress state of the glass, thereby further reducing the residual stress. Finally, when the quenching time increased to 14 s, the mean residual stress increased to 26.78 MPa (No. 7), indicating that the residual stress did not continue to decrease after the quenching time was further extended, but instead exhibited a certain fluctuation. This may have been because, after an excessively long quenching time, the temperature gradient between the glass surface and interior and the stress evolution process changed, so the residual stress release effect of the glass no longer continued to increase.

### 4.3. Effects of Process Parameters on Springback Displacement

According to the mean response table in Table 9, heating temperature (*A*) had a relatively significant effect on the mean springback displacement, with a range value of 4.066, indicating that, within the scope of this study, variations in heating temperature had a pronounced regulatory effect on the mean springback displacement. The range value of quenching air velocity (*C*) was 2.921, suggesting that it also had a noticeable effect on the mean springback displacement. The range value of quenching time (*E*) was 1.557, indicating that it had a certain influence on the mean springback displacement. In contrast, the range values of quenching air pressure (*D*) and holding time (*B*) were 1.287 and 0.446, respectively, showing that their effects on the mean springback displacement were relatively small. Within the selected range of process parameters, heating temperature (*A*), quenching air velocity (*C*), and quenching time (*E*) had more pronounced effects on the mean springback displacement, whereas the effects of quenching air pressure (*D*) and holding time (*B*) were relatively limited.

**Figure 14:**
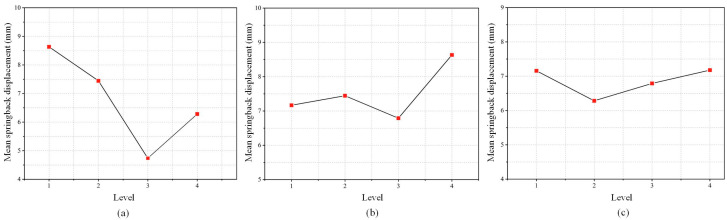
Main effects plot of process parameters on the mean springback displacement of glass: (**a**) heating temperature; (**b**) quenching air velocity; (**c**) quenching time.

As shown in Figure 14a, the mean springback displacement first decreased and then slightly increased with increasing heating temperature. When the temperature was 672.5 °C, the mean springback displacement was 8.635 mm (No. 3). As the temperature increased to 675 °C, the mean springback displacement decreased to 7.445 mm (No. 6). When the temperature further increased to 677.5 °C, the mean springback displacement further decreased to 4.736 mm (No. 9). However, when the temperature increased to 680 °C, the mean springback displacement slightly increased to 6.283 mm (No. 15). This indicated that, as the temperature increased, the softening effect of the glass was enhanced in the early stage, resulting in a significant reduction in springback displacement. However, at higher temperatures, excessive softening of the glass may have led to nonuniform deformation, causing a slight increase in springback displacement.

As shown in Figure 14b, the mean springback displacement exhibited a trend of first increasing slightly, then decreasing, and then increasing again as the quenching air velocity increased. When the quenching air velocity was 10 m/s, the mean springback displacement was 7.167 mm (No. 7). When the quenching air velocity increased to 12 m/s, the mean springback displacement increased significantly to 7.445 mm (No. 6). However, as the quenching air velocity further increased to 13 m/s, the mean springback displacement instead decreased to 6.79 mm (No. 8), and when the quenching air velocity reached 14 m/s, the mean springback displacement increased again to 8.635 mm (No. 3). This trend indicated that a moderate quenching air velocity was beneficial for reducing springback displacement, whereas excessively high or low air velocities may have caused fluctuations in springback displacement. In particular, when the air velocity was too high, the temperature difference between the glass surface and interior became excessively large, preventing thermal stress from being released uniformly, thereby increasing springback displacement and affecting forming quality.

As shown in Figure 14c, the mean springback displacement showed a trend of first decreasing and then increasing with increasing quenching time. When the quenching time was 8 s, the mean springback displacement was 7.157 mm (No. 16). When the quenching time was extended to 10 s, the mean springback displacement decreased to 6.283 mm (No. 15). When the quenching time further increased to 12 s, the mean springback displacement was 6.79 mm (No. 8). However, when the quenching time reached 14 s, the mean springback displacement increased to 7.171 mm (No. 10). A shorter quenching time may have led to a larger temperature difference between the glass surface and interior, thereby resulting in a higher springback displacement. As the quenching time increased, the internal temperature difference in the glass gradually decreased, leading to a reduction in springback displacement. However, beyond a certain point, an excessively long quenching time may have caused overcooling of the glass surface and a lag in internal temperature adjustment, resulting in an increase in springback displacement again.

## 5. Process Optimization

GMP involves a complex thermo-mechanical coupling process in which multiple process parameters interact nonlinearly to determine the final forming quality. Traditional trial-and-error methods are often inefficient and costly. To address this issue, a systematic two-step optimization strategy is proposed in this study. First, to overcome the high computational cost of iterative finite element simulations, the forming quality is rapidly characterized based on orthogonal experimental data. Subsequently, the NSGA-III multi-objective evolutionary algorithm is employed to efficiently search for optimal process parameter combinations, aiming to simultaneously minimize residual stress and springback displacement.

### 5.1. Model Development Based on Regression Analysis

In glass molding, product quality characteristics change significantly with variations in multiple control factors, and the relationship is not simply linear, making it difficult to obtain an analytical model. To address this issue, one approach is to establish the control factors in the response statistics based on regression analysis. The model can be expressed by Equation (1):

(1) $$y(x)=c_{0}+\sum_{i=1}^{n}c_{i}x_{i}+\sum_{ij(i<j)}^{n}c_{ij}x_{i}x_{j}$$

where *y* denotes the response function; *c*_0_, *c_i_*, and *c_ij_* denote the zero-order, first-order, and second-order coefficients, respectively; *x_i_* and *x_j_* denote the control factors; and *n* is the number of factors. Minitab 18 was used to develop the regression models for glass molding. The regression models for the mean residual stress (*R*_s_) and mean springback displacement (*S_d_*) are expressed in Equations (2) and (3), respectively, as follows:

(2) $$\begin{array}{ll} R_{s}= & 32.9+2.10A+11.74B-8.51C-6.97D+3.78E-0.237A^{2}-0.334B^{2}\\ & +1.36C^{2}+1.39D^{2}-0.414E^{2}-2.24AB+0.11AE-2.13BE+0.18CE \end{array}$$

(3) $$\begin{array}{ll} S_{d}= & 11.3-4.34A-7.86B+5.23C+2.63D-0.20E+0.439A^{2}+0.138B^{2}\\ & -0.67C^{2}-0.59D^{2}+0.356E^{2}+1.47AB-0.75AE+1.11BE-0.35CE \end{array}$$

The regression models developed in this study were constructed based on simulation data obtained from an L16(4^5) orthogonal experimental design and serve as response surface surrogate models to describe the relationship between process parameters and response variables, thereby providing an approximate representation of complex nonlinear relationships. To evaluate the predictive performance of the developed surrogate models, R^2^ was used as the evaluation metric. For the residual stress model, R^2^ = 0.9796; for the springback displacement model, R^2^ = 0.9297. The results indicate that the developed models show a reasonable level of agreement with the simulation data and may reflect the general nonlinear behavior of the response variables under varying process parameters.

### 5.2. Forming Quality Bi-Objective Optimization

To execute the actual optimization procedure, the regression models for the mean residual stress and mean springback displacement developed in Section 5.1 serve directly as the fitness functions to evaluate the forming quality. Meanwhile, the design variables are strictly constrained within the practical processing windows defined by the orthogonal experimental levels. Based on this formulation, NSGA-III is a reference-point-based multi-objective evolutionary algorithm that follows the NSGA-II framework. Its core idea is to emphasize the selection of individuals close to a predefined set of reference points while maintaining the nondominated characteristics of the population individuals [41,42]. NSGA-III can be used not only for multi-objective engineering optimization but also for bi-objective engineering parameter optimization. Therefore, in this study, NSGA-III was used to perform bi-objective optimization of the process parameters for automotive panoramic roof glass. The algorithm parameters were set as follows: the population size was 150, the maximum number of iterations was 600, the fitness function value deviation was 1 × 10^−100^, and the mutation probability was 0.0005.

**Figure 15:**
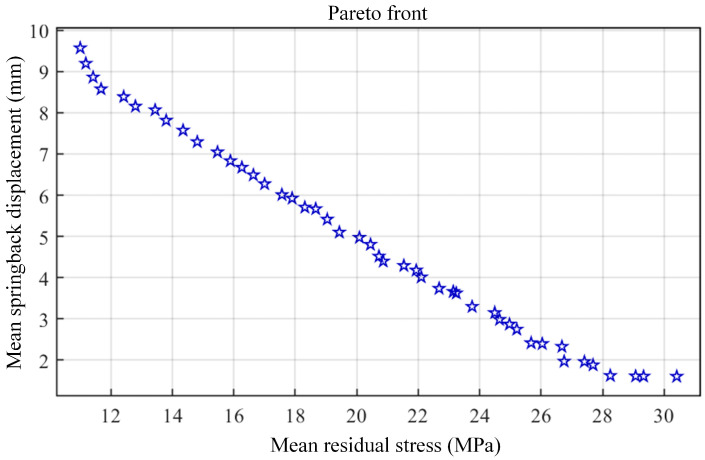
Pareto front solutions for bi-objective optimization.

As shown in Figure 15, the Pareto front obtained from the bi-objective optimization exhibited a relatively continuous and smooth distribution, indicating that the established optimization model could stably obtain the nondominated solution set between residual stress and springback displacement. In addition, there was a certain trade-off relationship between the two objectives; that is, the improvement of one objective was often accompanied by a change in the performance of the other, making it difficult for both objectives to reach their optimum simultaneously. The representative Pareto front solutions listed in Table 10 further reflected this characteristic. Different solutions corresponded to different process parameter combinations and also demonstrated the diversity of performance balance under different conditions. Based on the optimization results, a relatively obvious trade-off relationship existed between the mean residual stress and the mean springback displacement. Therefore, this study obtained a set of Pareto-optimal solutions for further comparison and selection, rather than a single absolutely optimal scheme. The obtained candidate solutions can be used for process parameter screening and scheme comparison, while their engineering applicability still needs to be further evaluated in future research through back-substitution analysis and field experiments.

### 5.3. Experimental Validation

**Figure 16:**
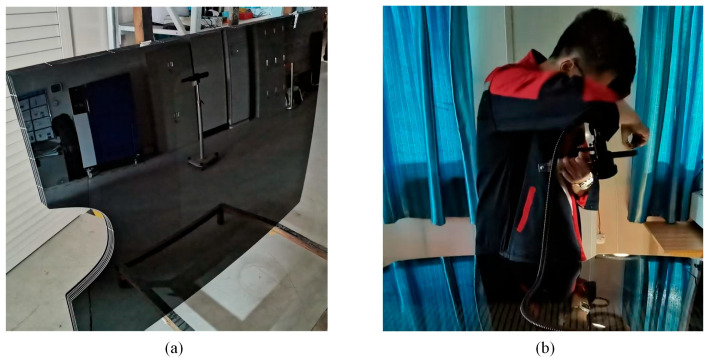
Formed panoramic roof and edge-stress measurement: (**a**) formed panoramic roof under different process conditions; (**b**) edge-stress measurement process.

Figure 16a shows the prepared panoramic roof specimens under different process parameter conditions, while Figure 16b presents the edge-stress measurement process performed by factory engineers using the VRP-100 edge stress meter (Strainoptics, Burnsville, MN, USA) for black-edge glass. The instrument adopts a reflective non-contact measurement method and is suitable for stress detection in the black-edge region of panoramic roofs. Both specimen preparation and stress measurement were conducted under actual manufacturing conditions, providing experimental support for subsequent process parameter optimization and model validation.

To preliminarily verify the applicability of the established model in predicting the mean residual stress and to account for the feasibility of the experimental measurement conditions, the mean residual stress was selected as the main experimental validation object in this study. On the one hand, the mean residual stress was one of the important statistical evaluation indicators used to characterize forming quality in this study. On the other hand, under the conditions of this study, the corresponding experimental data could be obtained through edge stress testing, and the mean residual stress could be further calculated statistically, thereby providing a basis for experimental validation of the optimization results. As shown in Table 11, for the three optimized parameter combinations obtained using the NSGA-III method, the relative errors between the experimentally measured mean residual stress and the predicted results were 13.7%, 9.3%, and 10.6%, respectively. Overall, the relative errors of the three optimized solutions were all controlled within 15%, indicating that the optimization results obtained by the NSGA-III method showed good consistency with the experimental results in terms of both variation trend and numerical level. This also indicated that the established multi-objective optimization model could reasonably reflect the relationship between process parameters and the mean residual stress, demonstrating certain feasibility and application value. It should be noted that the experimental validation in this study focused primarily on residual stress. This study was conducted in collaboration with an automotive company, and the industrial partner’s primary concern in glass manufacturing processes is related to residual-stress-induced quality issues. Springback displacement, while analyzed and optimized in the simulations, has not yet been experimentally validated. Future experimental validation of springback displacement will be carried out using 3D scanning or coordinate-based deviation mapping to quantitatively measure the final geometry, thereby further improving the predictive accuracy of the model. Although certain deviations still existed between the predicted and experimental results, the overall errors were within an acceptable range. Therefore, future studies may further validate the optimization scheme by expanding the sample dataset and incorporating more experimental results, so as to more comprehensively evaluate the accuracy and engineering applicability of the model.

## 6. Discussion

This study analyzed the effects of key process parameters during the GMP of automotive panoramic roofs and discussed the response characteristics of the stress field and displacement field of the glass during the quenching stage. The stress-field results showed that high-stress regions were mainly concentrated in geometrically discontinuous areas, such as groove transition zones and edges, indicating that local geometric features were highly sensitive to thermal stress distribution. Meanwhile, the U3 displacement-field results showed that the responses of the central and edge regions of the glass during quenching were not completely consistent, reflecting the combined effects of temperature gradients, local shrinkage behavior, and deformation readjustment after mold opening on the final shape.

The orthogonal analysis results further showed that different process parameters had different degrees of influence on the mean residual stress and mean springback displacement. Within the selected parameter range, heating temperature had a pronounced effect on both indicators, indicating that temperature conditions played an important role in determining the softening degree of glass, its stress release capacity, and subsequent springback behavior. Holding time and quenching time had relatively pronounced effects on the mean residual stress, whereas quenching air velocity had a more significant effect on the mean springback displacement. This indicated that, during process regulation, residual stress and springback displacement did not follow exactly the same variation pattern, and their dominant controlling factors differed to some extent. Therefore, in actual process design, different parameters should be targeted and matched according to quality-control objectives [43], rather than relying only on the adjustment of a single parameter to improve multiple performance indicators simultaneously.

In terms of parameter screening and validation, the candidate parameter combinations obtained based on the regression models and NSGA-III method showed relative errors within 15% for both indicators after simulation validation, indicating that the established response relationships could reflect the variation patterns between process parameters and quality indicators to some extent. However, it should also be noted that the current study was still mainly based on simulation analysis, and the amount of sample data was relatively limited, which may have affected the prediction accuracy in some parameter regions. Therefore, future studies could further increase the number of sample points, improve the model coverage of the design space, and validate key parameter combinations using experimental results, thereby further improving model accuracy and engineering application reliability. In addition, advances in manufacturing technologies may provide new perspectives for the fabrication of complex inorganic-material structures and related studies [44,45].

## 7. Conclusions

For the control of residual stress and springback displacement during the GMP of automotive panoramic roofs, this study established a corresponding finite element model and combined orthogonal experiments, regression analysis, and parameter screening methods to investigate the relationship between process parameters and precision forming quality indicators. Through numerical simulation, the variation characteristics of stress distribution and displacement response of the glass during the quenching stage were analyzed, the effects of different process parameters on the mean residual stress and mean springback displacement were revealed, and the screened parameter combinations were further validated. The main conclusions are as follows:

(1) A full-process finite element model for the GMP of automotive panoramic roofs was established. The model covered key stages including heating, press forming, pressure holding, mold opening, quenching, and slow cooling. Combined with temperature-dependent elastic modulus data and the generalized Maxwell viscoelastic model, the evolution of the temperature field, stress field, and displacement field of the glass throughout the forming process was numerically simulated. The results suggested that, during the quenching stage, different parameter combinations could affect the internal stress distribution and displacement response of the glass, with high-stress regions mainly concentrated in geometrically discontinuous areas such as groove transition zones and edges.
(2) For five key process parameters, namely heating temperature, holding time, quenching air velocity, quenching air pressure, and quenching time, simulation analysis was carried out using an L16(4^5) orthogonal design, and the effects of different parameter combinations on the mean residual stress and mean springback displacement were systematically investigated. The results showed that, within the given parameter range, heating temperature, holding time, and quenching time had relatively pronounced effects on the mean residual stress. The mean residual stress was relatively low when the heating temperature was 680 °C, the holding time was 3 s, and the quenching time was 12 s. Meanwhile, heating temperature, quenching air velocity, and quenching time had relatively more significant effects on the mean springback displacement. The mean springback displacement was relatively low when the heating temperature was 677.5 °C, the quenching air velocity was 13 m/s, and the quenching time was 10 s.
(3) Based on the established regression models, the process parameter combinations were further screened, and several candidate optimization schemes were obtained using the NSGA-III method. The experimental validation results showed that the relative errors of the mean residual stress corresponding to the optimized schemes were all controlled within 15%, indicating that the established model could reasonably reflect the relationship between process parameters and precision forming quality indicators, and that the obtained parameter schemes had certain feasibility. Although the present study has provided some useful insights, the model can still be further refined to improve its predictive capability and engineering applicability. For example, the viscoelastic parameters used in the generalized Maxwell model were obtained from referenced creep data rather than direct measurements on the specific panoramic-roof glass. Therefore, future work may consider conducting direct creep and stress-relaxation tests and further evaluating the possible influence of viscoelastic parameter uncertainty on residual-stress and springback predictions. Considering that the current study still had a relatively limited number of samples, future work may further validate the optimization schemes by expanding the dataset and incorporating more experimental results, thereby continuously improving the prediction accuracy of the model and the reliability of its engineering application, and providing guidance for precision forming and process optimization of large-scale thin-walled automotive panoramic roofs.

## Figures and Tables

**Table 1:** Material parameters of panoramic roof glass [21].

Properties	Value
Density (g/cm^3^)	2.5
Poisson’s ratio	0.2
Thermal conductivity (W/m·K)	1
Specific heat capacity (J/(kg·K))	880
Coefficient of thermal expansion (/°C) (20~300 °C)	8.75 × 10^−6^

**Table 2:** Comparison of elastic modulus fitting algorithms.

Model	R^2^	RMSE	MAE
Cubic polynomial	0.9807	655.33	638.39
Gaussian process regression	0.9731	772.66	595.17
Smoothing spline	0.9928	400.95	297.98
Double exponential	0.9996	96.7026	71.8712

**Table 3:** Variation of the elastic modulus of glass with temperature.

Temperature (°C)	Value (MPa)	Temperature (°C)	Value (MPa)
25	72,630	400	68,090
100	71,722	450	67,304.21
150	71,130.75	500	66,240
250	69,968.53	550	63,460
300	69,300.67	590	59,499.62
350	68,782.28	600	57,950

**Table 4:** Comprehensive creep test data of panoramic roof glass [21].

Time (s)	Normalized Shear Compliance *j_s_*	Normalized Bulk Compliance *j_k_*	Time (s)	Normalized Shear Compliance *j_s_*	Normalized Bulk Compliance *j_k_*
0.01	1.35	1.35	1.5	3.52	3.52
0.02	1.37	1.37	2.0	3.98	3.98
0.04	1.4	1.4	2.5	4.03	4.03
0.1	1.89	1.89	3.0	4.15	4.15
0.2	2.03	2.03	3.5	4.26	4.26
0.5	2.72	2.72	4.0	4.42	4.42
0.8	3.25	3.25	4.5	4.55	4.55
1.0	3.46	3.46	5.0	4.65	4.65

**Table 5:** Control factors and standard settings of levels.

Level	Control Factors				
	*A* (°C)	*B* (s)	*C* (m/s)	*D* (MPa)	*E* (s)
1	672.5	0.5	10	2	8
2	675	0.8	12	3	10
3	677.5	1.5	13	4	12
4	680	3	14	5	14

**Table 6:** Orthogonal simulation experiment design.

No.	Control Factors				
	*A* (°C)	*B* (s)	*C* (m/s)	*D* (MPa)	*E* (s)
1	672.5	0.5	10	2	8
2	672.5	0.8	13	5	10
3	672.5	1.5	14	3	12
4	672.5	3	12	4	14
5	675	0.5	14	4	10
6	675	0.8	12	3	8
7	675	1.5	10	5	14
8	675	3	13	2	12
9	677.5	0.5	12	5	12
10	677.5	0.8	14	2	14
11	677.5	1.5	13	4	8
12	677.5	3	10	3	10
13	680	0.5	13	3	14
14	680	0.8	10	4	12
15	680	1.5	12	2	10
16	680	3	14	5	8

**Table 7:** Response table of the orthogonal simulation experiments.

No.	Mean Residual Stress (MPa)	Mean Springback Displacement (mm)	No.	Mean Residual Stress (MPa)	Mean Springback Displacement (mm)
1	33.18	7.361	9	27.91	4.736
2	32.27	7.69	10	23.67	7.177
3	30.62	8.635	11	26.75	3.944
4	23.82	11.149	12	26.23	2.715
5	26.01	9.745	13	22.26	4.681
6	27.71	7.445	14	22.96	3.786
7	26.78	7.167	15	20.48	6.283
8	25.4	6.79	16	21.08	7.157

**Table 8:** Mean response table of stress for panoramic roof glass.

Level	*A* (°C)	*B* (s)	*C* (m/s)	*D* (MPa)	*E* (s)
1	29.97	27.34	27.29	25.68	27.18
2	26.48	26.65	24.98	26.71	26.25
3	26.14	26.16	26.67	24.88	26.72
4	21.7	24.13	25.35	27.01	24.13
Delta	8.28	3.23	2.31	2.13	3.05
Order	1	2	5	4	3

**Table 9:** Mean response table of springback displacement for panoramic roof glass.

Level	*A* (°C)	*B* (s)	*C* (m/s)	*D* (MPa)	*E* (s)
1	8.709	6.631	5.258	6.903	6.477
2	7.787	6.525	7.403	5.869	6.608
3	4.643	6.507	5.776	7.156	5.987
4	5.477	6.953	8.178	6.688	7.543
Delta	4.066	0.446	2.921	1.287	1.557
Order	1	5	2	4	3

**Table 10:** Representative Pareto front solutions for bi-objective optimization.

No.	Control Factors					Mean Residual Stress (MPa)	Mean Springback Displacement (mm)
	*A* (°C)	*B* (s)	*C* (m/s)	*D* (MPa)	*E* (s)		
1	672.55	2.95	11.76	4.01	9.89	36.54	1.910
2	674.37	2.87	11.11	3.39	9.75	32.53	2.208
3	676.23	2.99	10.62	4.64	10.6	27.62	2.764
4	678.14	2.98	10.23	4.37	8.98	26.75	1.969
5	679.48	2.65	10.34	4.40	13.22	11.67	8.785
6	676.94	0.54	10.06	4.69	9.04	30.21	3.872
7	677.49	1.12	10.02	3.57	9.22	28.70	3.634
8	677.25	1.75	10.01	3.83	10.95	27.14	3.872
9	679.28	2.98	10.32	4.42	9.71	20.72	4.516

**Table 11:** Comparison between optimized predicted results and experimental results.

Group	Control Factors					Experimental Results	Relative Error
	*A* (°C)	*B* (s)	*C* (m/s)	*D* (MPa)	*E* (s)	*R_s_* (MPa)	*R_s_* (%)
3	676.23	2.99	10.62	4.64	10.6	31.4	13.7
7	677.49	1.12	10.02	3.57	9.22	31.37	9.3
9	679.28	2.98	10.32	4.42	9.71	22.91	10.6

## Data Availability

The original contributions presented in this study are included in the article. Further inquiries can be directed to the corresponding authors.

## References

[1] Ojovan M.I., Darmaev M.V., Mashanov A.A., Ojovan M.I., Darmaev M.V., Mashanov A.A. (2025). Advances in Glass and Glass Crystalline Materials: An Overview. AIMS Mater. Sci..

[2] Almendro-Candel M.B., Vidal M.M.J. (2024). Glasses, Frits and Glass-Ceramics: Processes and Uses in the Context of Circular Economy and Waste Vitrification. Coatings.

[3] Yang Y., Tang Y., Jiang G., Fang G., Xue Y., Tan Z., Zhang X., Pan D. (2025). Advances in Glass-Ceramics Raw Material Selection-Performance Modulation-Application Expression: A Review. Mater. Today.

[4] Nimbalkar P., Bhaskar P., Kumar L.N.V., Narayanan M., Torres E., Venkataramanan S.S.A., Kathaperumal M. (2025). A Review of Glass Substrate Technologies. Chips.

[5] Michael M., Favoino F., Jin Q., Luna-Navarro A., Overend M. (2023). A Systematic Review and Classification of Glazing Technologies for Building Façades. Energies.

[6] Righini G.C., Ferrari M., Lukowiak A., Macrelli G. (2025). Flexible Glass: Myth and Photonic Technology. Materials.

[7] Wu S., Sun H., Duan M., Mao H., Wu Y., Zhao H., Lin B. (2023). Applications of Thermochromic and Electrochromic Smart Windows: Materials to Buildings. Cell Rep. Phys. Sci..

[8] Allsopp B.L., Orman R., Johnson S.R., Baistow I., Sanderson G., Sundberg P., Stålhandske C., Grund L., Andersson A., Booth J. (2020). Towards Improved Cover Glasses for Photovoltaic Devices. Prog. Photovolt. Res. Appl..

[9] Tuck E.O., Stokes Y.M., Schwartz L.W. (1997). Slow Slumping of a Very Viscous Liquid Bridge. J. Eng. Math..

[10] Stokes Y.M. (2000). Numerical Design Tools for Thermal Replication of Optical-Quality Surfaces. Comput. Fluids.

[11] Hunt R. (2002). Numerical Solution of the Flow of Thin Viscous Sheets under Gravity and the Inverse Windscreen Sagging Problem. Int. J. Numer. Methods Fluids.

[12] Parsa M.H., Rad M., Shahhosseini M.R., Shahhosseini M.H. (2005). Simulation of Windscreen Bending Using Viscoplastic Formulation. J. Mater. Process. Technol..

[13] Ananthasayanam B., Joseph P.F., Joshi D., Gaylord S., Petit L., Blouin V.Y., Richardson K.C., Cler D.L., Stairiker M., Tardiff M. (2012). Final Shape of Precision Molded Optics: Part I—Computational Approach, Material Definitions and the Effect of Lens Shape. J. Therm. Stress..

[14] Hung C.-H., Hung S.-Y., Shen M.-H., Yang H. (2012). Semiellipsoid Microlens Fabrication Method Using the Lift-off and Alignment Exposure Processes. J. Micromech. Microeng..

[15] Shutov A.I., Popov P.V., Kolos A.P. (2001). Testing of Hardened Glass for the Type of Fracture. Glass Ceram..

[16] Shutov A.I., Novikov I.A., Ostapko A.S. (2002). Prospects of a New Method for Thermal Treatment of Sheet Glass. Glass Ceram..

[17] Shutov A.I., Ostapko A.S., Ostapko T.S. (2003). The Effect of Complex Thermal Treatment Parameters on Properties of Sheet Glass. Glass Ceram..

[18] Vu A.T., Meiners C., Bögershausen S., Rojacher C., Brepols T., Reese S., Bergs T. Glass4AutoFuture: Modeling Thermal-Mechanical Dynamics in Vacuum-Assisted Deep Drawing of 3D Thin Glass Components for Automotive Interiors. Proceedings of the 28th International ESAFORM Conference on Material Forming.

[19] Corre B.L., Collin A., Soudre-Bau L., Meshaka Y., Jeandel G. (2014). Glass Sagging Simulation with Improved Calculation of Radiative Heat Transfer by the Optimized Reciprocity Monte Carlo Method. Int. J. Heat Mass Transf..

[20] Nielsen J.H., Olesen J.F., Poulsen P.N., Stang H. (2010). Finite Element Implementation of a Glass Tempering Model in Three Dimensions. Comput. Struct..

[21] Zhang L. (2022). Die Design and Forming Process Optimization for Hot-Press Forming of Automotive Rear Windshield Glass. Master’s Thesis.

[22] Iglesias A., Martinez-Agirre M., Torca I., Llavori I., Esnaola J.A. (2022). Numerical Methodology Based on Fluid-Structure Interaction to Predict the Residual Stress Distribution in Glass Tempering Considering Non-Uniform Cooling. Comput. Struct..

[23] Fu H., Xue C., Liu Y., Cao B., Lang C., Yang C. (2022). Prediction Model of Residual Stress during Precision Glass Molding of Optical Lenses. Appl. Opt..

[24] Chen Z., Hu S., Zhang S., Zhang Q., Zhang Z., Ming W. (2023). Simulation and Experimental Study on the Precision Molding of Irregular Vehicle Glass Components. Micromachines.

[25] Yang W., Zhang Z., Ming W., Yin L., Zhang G. (2022). Study on Shape Deviation and Crack of Ultra-Thin Glass Molding Process for Curved Surface. Ceram. Int..

[26] Zhao X., Hu S., Sun P., Ming W. (2025). Precision Molding Simulation Study of 3D Ultra-Thin Glass Components for Smartwatches. Micromachines.

[27] Yao H., Lv K., Zhang J., Wang H., Xie X., Zhu X., Deng J., Zhuo S. (2019). Effect of Elastic Modulus on the Accuracy of the Finite Element Method in Simulating Precision Glass Molding. Materials.

[28] Shu C., Yin S., Li Y., Mao Z., Guo X., Huang S. (2022). High-Precision Molding Simulation Prediction of Glass Lens Profile for a New Lanthanide Optical Glass. Ceram. Int..

[29] Luo H., Yu J., Hu J., Tang K., Xu B., Wang F. (2022). Effects of Uniform/Nonuniform Interface Friction on Mold-Filling Behavior of Glass Microarray: A Numerical-Experimental Study. Tribol. Lett..

[30] Chen Y., Zhang S., Hu S., Zhao Y., Zhang G., Cao Y., Ming W. (2023). Study of Heat Transfer Strategy of Metal Heating/Conduction Plates for Energy Efficiency of Large-Sized Automotive Glass Molding Process. Metals.

[31] Huang S., Jiang C., Tian Z., Ren B., Tang Y., Xie F., Zheng Y., Gao Q. (2023). Optimization of Precision Molding Process Parameters of Viscoelastic Materials Based on BP Neural Network Improved by Genetic Algorithm. Mater. Today Commun..

[32] Mikulikova A., Mesicek J., Karger J., Hajnys J., Ma Q.-P., Sliva A., Smiraus J., Srnicek D., Cienciala S., Pagac M. (2023). Topology Optimization of the Clutch Lever Manufactured by Additive Manufacturing. Materials.

[33] Chai B.X., Eisenbart B., Nikzad M., Fox B., Blythe A., Blanchard P., Dahl J. (2023). A Novel Heuristic Optimisation Framework for Radial Injection Configuration for the Resin Transfer Moulding Process. Compos. Part A.

[34] Purr S., Wendt A., Meinhardt J., Moelzl K., Werner A., Hagenah H., Merklein M. (2016). Data-Driven Inline Optimization of the Manufacturing Process of Car Body Parts. IOP Conf. Ser. Mater. Sci. Eng..

[35] Wang L.-C., Chen C.-C., Hsu C.-C. (2022). Applying Machine Learning and GA for Process Parameter Optimization in Car Steering Wheel Manufacturing. Int. J. Adv. Manuf. Technol..

[36] Jankovics D., Barari A. (2019). Customization of Automotive Structural Components Using Additive Manufacturing and Topology Optimization. IFAC-Pap..

[37] Liu X., Zhou J., Tao B., Shu Y., Feng Z., Chen S.-C., Zhang Y., Yi A.Y. (2026). Precision Glass Aspherical Lens Manufacturing by Compression Molding: A Review. Light. Adv. Manuf..

[38] Ming W., Chen Z., Du J., Zhang Z., Zhang G., He W., Ma J., Shen F. (2020). A Comprehensive Review of Theory and Technology of Glass Molding Process. Int. J. Adv. Manuf. Technol..

[39] Zhang L., Liu W. (2017). Precision Glass Molding: Toward an Optimal Fabrication of Optical Lenses. Front. Mech. Eng..

[40] Novikov V.N., Sokolov A.P. (2022). Temperature Dependence of Structural Relaxation in Glass-Forming Liquids and Polymers. Entropy.

[41] Deb K., Jain H. (2014). An Evolutionary Many-Objective Optimization Algorithm Using Reference-Point-Based Nondominated Sorting Approach, Part I: Solving Problems With Box Constraints. IEEE Trans. Evol. Comput..

[42] Ishibuchi H., Pang L.M., Gong C. (2025). Search Behavior Analysis of NSGA-III: Dominance-Based and Decomposition-Based Multi-Objective Evolutionary Algorithm. Proceedings of the Genetic and Evolutionary Computation Conference.

[43] Ming W. (2026). State of the Art of the Role of Artificial Intelligence in Electrical Discharge Machining: A Review. Eng. Appl. Artif. Intell..

[44] Duan L., Bai M., Xiao N., Zhang L., Wang H., Wu Y., Dong B., Tang W., Li Y., Zhang Z. (2026). A Review of Recent Advances in Ceramic 3D Printing Technologies. J. Manuf. Process..

[45] Xin C., Li Z., Hao L., Li Y. (2023). A Comprehensive Review on Additive Manufacturing of Glass: Recent Progress and Future Outlook. Mater. Des..

